# Impaired sensitivity to thyroid hormones is associated with atrial fibrillation in the euthyroid population

**DOI:** 10.3389/fcvm.2026.1674778

**Published:** 2026-03-24

**Authors:** Han Gong, Chun Zhou, Yuanjun Sun, Chengming Ma, Zhipeng Zhang, Yang Liu, Xiaomeng Yin

**Affiliations:** 1 Department of Cardiology, The First Affiliated Hospital of Dalian Medical University, Dalian, China; 2 Institute of Cardiovascular Diseases, The First Affiliated Hospital of Dalian Medical University, Dalian, China

**Keywords:** euthyroid population, paroxysmal atrial fibrillation, persistent atrial fibrillation, sensitivity to thyroid hormones, thyroid hormones

## Abstract

**Background:**

The relationship between thyroid hormone sensitivity and atrial fibrillation (AF) progression remains unclear.

**Objective:**

This study aimed to investigate the relationship between impaired thyroid hormone sensitivity and the progression of AF in individuals with normal thyroid function.

**Methods:**

This retrospective cross-sectional study included 1,190 consecutive patients with AF, who were classified into paroxysmal AF (*n* = 623) and persistent AF (*n* = 567) groups. Data on cardiac ultrasound and serum biochemical parameters were obtained. Thyroid hormone sensitivity was assessed using a parametric thyroid feedback quantile-based index (PTFQI), thyrotropin-thyroxine resistance index (TT4RI), and peripheral free triiodothyronine/free thyroxine (FT3/FT4) ratio. Logistic regression analysis was performed to assess the relationship between thyroid hormone sensitivity indices and AF classification.

**Results:**

Compared with the paroxysmal AF group, patients with persistent AF exhibited significantly higher PTFQI and TT4RI values and a lower FT3/FT4 ratio (*P* for trend <0.001). In the multivariable logistic regression analysis, persistent AF was independently and significantly associated with elevated TT4RI and PTFQI as well as a reduced FT3/FT4 ratio (all *P* for trend <0.05). In the fully adjusted model, the odds ratios (95% confidence intervals) for TT4RI, PTFQI, and FT3/FT4 ratios were 1.038 (1.010–1.066), 4.063 (1.527–10.809), and 0 (0–0.071), respectively. Quartile-based analysis further revealed a consistent and significant association between higher PTFQI scores and the probability of persistent AF across all upper quartiles (*P* < 0.01). The FT3/FT4 ratio demonstrated a non-linear association with persistent AF, acting as a risk factor in the third quartile (Q3, *P* for trend = 0.018) and as a protective factor in the fourth quartile (Q4, *P* for trend = 0.003). No statistically significant differences were observed between TT4RI quartiles.

**Conclusion:**

Impaired thyroid hormone sensitivity may contribute to the progression from paroxysmal to persistent AF in euthyroid patients; however, its clinical predictive value requires further validation in large-scale studies.

## 1 Introduction

Atrial fibrillation (AF) is a chronic progressive arrhythmia characterized by the replacement of regular atrial electrical activity with rapid and disorganized fibrillatory waves. It is one of the most common arrhythmias observed in clinical practice. In addition to the symptoms induced by arrhythmia, AF significantly increases the risk of stroke, heart failure, cognitive impairment, and all-cause mortality, thereby contributing to higher disability rates and reduced quality of life in affected patients (1–3).

Conversely, endocrine disorders are recognized as potential etiological factors for the development of AF. Thyroid hormones have a significant impact on the heart by exerting cardioprotective effects through the promotion of cardiomyocyte proliferation and neovascularization (4, 5). A randomized controlled trial reported that, in patients with acute anterior ST-segment elevation myocardial infarction (STEMI), short-term LT3 therapy was associated with improved left ventricular dilation parameters at discharge and a trend toward reduced infarct size during follow-up, suggesting a potential role of thyroid hormones in post-infarction cardiac remodeling (6). However, excessive thyroid hormone levels may impair cardiac function (7). In thyrotoxicosis, shortening of the atrial action potential duration and cardiac structural remodeling, including fibrosis, may promote AF development (8, 9). Numerous studies have demonstrated a robust association between hyperthyroidism and AF (10–12). Subclinical thyroid dysfunction is also associated with AF incidence (13). In recent years, central thyroid hormone sensitivity indices, such as the parametric thyroid feedback quantile-based index (PTFQI), thyrotropin-thyroxine resistance index (TT4RI), and peripheral index free triiodothyronine/free thyroxine (FT3/FT4) ratio, have offered new insights into the evaluation of tissue-level effects of thyroid hormones in individuals with normal thyroid function (14). Emerging evidence indicates that impaired thyroid hormone sensitivity may significantly contribute to the development and progression of AF, even in euthyroid individuals (15, 16). However, the role of thyroid hormone sensitivity in the staging of AF and its progression to persistent AF has not been systematically investigated.

Therefore, this study aimed to investigate the relationship between thyroid hormone sensitivity indices (TT4RI, PTFQI, and FT3/FT4 ratio) and AF type, exploring their association with AF subtype and their potential role in AF progression from paroxysmal to persistent forms, as well as their clinical implications for AF management.

## 2 Methods

### 2.1 Study design and participants

**Figure 1:**
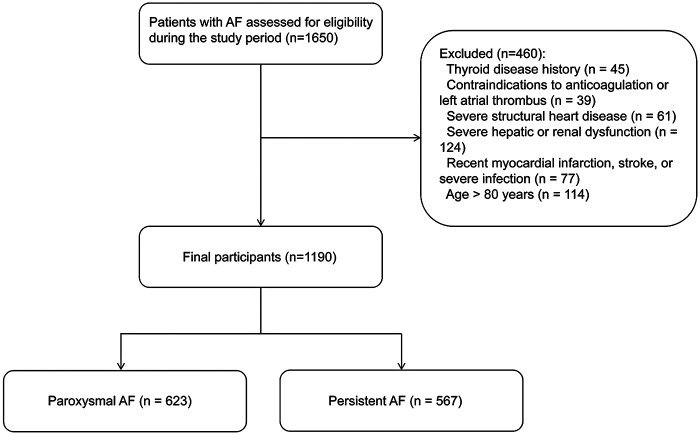
Flowchart of patient enrollment and study population.

This was a single-center retrospective cross-sectional study. Patients diagnosed with paroxysmal or persistent AF who were confirmed to have normal thyroid function between January 2023 and January 2024 at the First Affiliated Hospital of Dalian Medical University were included in this study. The inclusion criteria were as follows: (1) age between 18 and 80 years; (2) diagnosis of AF confirmed by 12-lead electrocardiogram (ECG), 24 h ambulatory ECG, or clinical records; (3) paroxysmal AF, defined as AF episodes lasting <7 days and terminating spontaneously or within 48 h; and (4) persistent AF, defined as episodes lasting >7 days. Patients were excluded if they had the following conditions: (1) a current or previous history of thyroid disease such as hyperthyroidism, hypothyroidism, subclinical thyroid dysfunction, or had undergone thyroidectomy; (2) contraindications to anticoagulant therapy; (3) a thrombus in the left atrium; (4) severe structural heart disease, including moderate to severe valvular disease and hypertrophic or dilated cardiomyopathy; (5) severe hepatic or renal dysfunction; or (6) a recent (one month) history of acute myocardial infarction, stroke, or severe infection. Eligible patients were categorized into the paroxysmal or persistent AF groups based on their arrhythmia type. The detailed screening process is presented in Figure 1. This study was approved by the Ethics Committee of the First Affiliated Hospital of Dalian Medical University (Approval No. PJ-KS-KY-2025-583).

### 2.2 Data collection

General clinical data were obtained for all patients, including sex, age, history of smoking, alcohol consumption, hypertension, diabetes mellitus, stroke, coronary artery disease, and heart failure as documented during hospitalization. Serum biochemical parameters included thyroid-stimulating hormone (TSH), FT3, FT4, low-density lipoprotein cholesterol (LDL-C), high-density lipoprotein cholesterol (HDL-C), triglyceride (TG), total cholesterol (TC), N-terminal pro-B-type natriuretic peptide (NT-proBNP), and creatinine (CRE). All blood samples were collected from fasting venous blood in the morning, following admission to the hospital. Serum TSH, FT3 and FT4 were measured in the Department of Nuclear Medicine of our hospital using an automated chemiluminescent immunoassay under standardized internal quality control procedures. The reference ranges were 0.35–5.10 μIU/mL for TSH, 2.76–6.45 pmol/L for FT3, and 11.2–23.81 pmol/L for FT4. Other biochemical parameters were measured using routine automated assays in the hospital clinical laboratory. Transthoracic echocardiography was performed during hospitalization to assess cardiac structure and function, and the following parameters were recorded: left atrial diameter (LAD), left ventricular end-diastolic diameter (LVID), and left ventricular ejection fraction (LVEF).

### 2.3 Definitions and calculations of thyroid hormone sensitivity

Thyroid hormone sensitivity includes both central and peripheral hormone sensitivity indices. The central thyroid hormone sensitivity indices included the PTFQI and TT4RI. TT4RI was calculated as TT4RI = FT4 (pmol/L) × TSH (mIU/L), and higher values indicate reduced central sensitivity. The PTFQI is a new index with values ranging from −1 to +1, where negative values signify heightened sensitivity and positive values signify pituitary resistance. It was calculated as: PTFQI = F [(FT4 − µFT4)/*σ*FT4] − {1 − F [(ln TSH − µLn TSH)/*σ*ln TSH]}. Here, F denotes the cumulative distribution function of the standard normal distribution. The population mean (μ) and standard deviation (*σ*) for the Chinese population were as follows: μFT4 = 16.3802, *σ*FT4 = 1.98049, μln TSH = 0.5865, and *σ*ln TSH = 0.43854 (17). The peripheral thyroid hormone sensitivity index was calculated using the following formula: FT3/FT4 ratio = FT3 (pmol/L)/FT4 (pmol/L), where a lower FT3/FT4 ratio indicates poorer peripheral sensitivity to thyroid hormones (18).

### 2.4 Statistical analyses

Statistical analyses were performed using Statistical Package for the Social Sciences software (version 27.0; IBM Corp., Armonk, NY, USA). The Kolmogorov–Smirnov test was used to assess the normality of the continuous variables. Normally distributed data are presented as mean ± standard deviation and compared using the independent samples *t*-test, whereas non-normally distributed data are expressed as median and interquartile range and compared using the Mann–Whitney *U-*test. Categorical variables were summarized as frequencies and percentages and were analyzed using the chi-square test. After testing for multicollinearity, logistic regression analyses were conducted to assess the relationship between the thyroid hormone sensitivity indices and AF type. Three models were constructed: Model 1, unadjusted; Model 2, adjusted for age and sex; Model 3, further adjusted for age, sex, smoking, alcohol consumption, hypertension, diabetes mellitus, stroke, coronary artery disease, heart failure, LDL-C, HDL-C, TG, NT-proBNP, CRE, LAD, LVID, and LVEF. Statistical significance was defined as a two-sided *P*-value of <0.05.

## 3 Results

### 3.1 Baseline characteristics of the study participants

The baseline characteristics of the study population are summarized in Table 1. A total of 1,190 patients were included in the study, comprising 739 men and 451 women. Compared with the patients in the paroxysmal AF group, those with persistent AF were older, more likely to consume alcohol (all *P* < 0.05), and exhibited higher rates of comorbidities, including stroke, coronary artery disease (CAD), and heart failure (all *P* < 0.05). With respect to echocardiographic parameters, patients with persistent AF exhibited significantly larger LAD and LVID, and lower LVEF (all *P* < 0.001). Biochemically, NT-proBNP, FT4, TSH, and CRE levels were significantly higher in the persistent AF group, whereas lipid parameters, including LDL-C, HDL-C, TG, and TC, were significantly lower (all *P* < 0.05). Regarding thyroid hormone sensitivity indices, TT4RI and PTFQI were significantly higher in patients with persistent AF, whereas the FT3/FT4 ratio was significantly lower than in patients with paroxysmal AF (all *P* < 0.001). No significant differences were observed between the two groups in terms of smoking history, hypertension, diabetes mellitus, or FT3 level.

**Table 1:** Baseline characteristics of the study population.

Characteristic	Overall (*n* = 1,190)	Paroxysmal AF (*n* = 623)	Persistent AF (*n* = 567)	*P*
Sex, *n* (%)				
Male	739 (62.1)	360 (57.8)	379 (66.8)	**<0**.**001**
Female	451 (37.9)	263 (42.2)	188 (33.2)	
Age	70 (64–77)	69 (62–76)	71 (65–78)	**0**.**005**
Drinking, *n* (%)				
No	980 (82.4)	528 (84.8)	452 (79.7)	**0**.**023**
Yes	210 (17.6)	95 (15.2)	115 (20.3)	
Smoking, *n* (%)				
No	885 (74.4)	476 (76.4)	409 (72.1)	0.092
Yes	305 (25.6)	147 (23.6)	158 (27.9)	
Hypertension, *n* (%)				
No	452 (38)	249 (40)	203 (35.8)	0.139
Yes	738 (62)	374 (60)	364 (64.2)	
Diabetes, *n* (%)				
No	880 (73.9)	468 (75.1)	412 (72.7)	0.335
Yes	310 (17.6)	155 (24.9)	155 (27.3)	
Stroke, *n* (%)				
No	952 (80)	529 (84.9)	423 (74.6)	**<0**.**001**
Yes	238 (20)	94 (15.1)	144 (25.4)	
CAD, *n* (%)				
No	684 (57.5)	377 (60.5)	307 (54.1)	**0**.**026**
Yes	506 (52.5)	246 (39.5)	260 (45.9)	
Heart Failure, *n* (%)				
No	744 (62.5)	506 (81.2)	238 (42)	**<0**.**001**
Yes	446 (37.5)	117 (18.8)	329 (58)	
LAD (mm)	40 (37–45)	38 (36–40)	44 (40–48)	**<0**.**001**
LVID (mm)	48 (45–52)	47 (44–50)	49 (46–54)	**<0**.**001**
LVEF (mm)	58 (54–59)	59 (57–59)	55 (45–59)	**<0**.**001**
NT-proBNP (ng/L)	69.34 (39.12–184.33)	51.5 (35.6–124)	124 (48.6–215.4)	**<0**.**001**
FT3 (pmol/L)	4.17 (15.78–18.56)	4.18 (3.83–4.59)	4.16 (3.84–4.51)	0.246
FT4 (pmol/L)	17.09 ± 2.06	16.64 ± 1.93	17.84 ± 2.00	**<0**.**001**
TSH (mIU/L)	1.81 (1.38–2.37)	1.66 (1.27–2.16)	2.05 (1.53–2.66)	**<0**.**001**
LDL-C (mmol/l)	2.34 (1.82–2.86)	2.4 (1.88–2.96)	2.26 (1.75–2.74)	**0**.**001**
HDL-C (mmol/l)	1.07 (0.91–1.26)	1.08 (0.92–1.27)	1.05 (0.88–1.25)	**0**.**034**
TG (mmol/l)	1.2 (0.89–1.62)	1.26 (0.95–1.68)	1.14 (0.84–1.56)	**0**.**003**
TC (mmol/l)	4.24 (3.54–4.98)	4.37 (3.67–5.10)	4.09 (3.45–4.80)	**<0**.**001**
Cre (mmol/l)	70 (60–83)	68 (58–82)	72 (61–84)	**0**.**005**
TT4RI	30.99 (23.43–39.92)	27.47 (21.13–34.65)	35.99 (27.18–45.78)	**<0**.**001**
PTFQI	0.09 (−0.15–0.38)	−0.06 (−0.29–0.19)	0.25 (0.02–0.50)	**<0**.**001**
FT3/FT4 ratio	0.24 (0.22–0.27)	0.25 (0.23–0.28)	0.23 (0.21–0.26)	**<0**.**001**

CAD, coronary artery disease; LAD, Left Atrial Diameter; LVID, left ventricular end-diastolic diameter; LVEF, Left Ventricular Ejection Fraction; NT-proBNP, N - terminal pro - B - type Natriuretic Peptide; FT3, Free Triiodothyronine; FT4, Free Thyroxine; TSH, Thyroid-Stimulating Hormone; LDL-C, Low-Density Lipoprotein Cholesterol; HDL-C, High-Density Lipoprotein Cholesterol; TG, Triglycerides; TC, Total Cholesterol; Cre, Creatinine; TT4RI, Thyrotropin Thyroxine Resistance Index; PTFQI, Parametric Thyroid Feedback Quantile - Based Index. The bold values indicate statistically significant *P*-values (*P* < 0.05).

### 3.2 Association of thyroid hormone sensitivity indices with AF progression

Multicollinearity was assessed before logistic regression analyses, which revealed significant collinearity between TC and LDL-C levels. Higher TT4RI and PTFQI levels were significantly associated with persistent AF, while a lower FT3/FT4 ratio was independently associated with persistent AF (all *P* < 0.05; Table 2). In the fully adjusted model (Model 3), which was adjusted for age, sex, smoking, alcohol consumption, hypertension, diabetes mellitus, stroke, CAD, heart failure, LAD, LVID, LVEF, NT-proBNP, LDL-C, HDL-C, TG, and CRE, the odds ratios (ORs) [95% confidence intervals (CIs)] for TT4RI, PTFQI, and FT3/FT4 ratio were 1.038 (1.010–1.066), 4.063 (1.527–10.809), and 0 (0–0.071), respectively (Table 2; all *P* for trend <0.05).

**Table 2:** Logistic regression analysis for the association of thyroid hormone sensitivity with persistent AF.

	Model 1		Model 2		Model 3	
Variable	OR (95%CI)	*P*	OR (95%CI)	*P*	OR (95%CI)	*P*
FT3/FT4 ratio	0 (0–0.0001)	<**0**.**001**	0 (0–0.0002)	<**0**.**001**	0 (0–0.071)	**0**.**004**
PTFQI	2.748 (1.309–5.767)	**0**.**008**	2.575 (1.191–5.566)	**0**.**016**	4.063 (1.527–10.809)	**0**.**005**
TT4RI	1.047 (1.025–1.069)	<**0**.**001**	1.049 (1.027–1.072)	<**0**.**001**	1.038 (1.010–1.066)	**0**.**005**

Model 1: Crude model. Model 2: Adjusted for sex and age. Model 3: Adjusted for age, sex, smoking, alcohol consumption, hypertension, diabetes mellitus, stroke, coronary artery disease, heart failure, LDL-C, HDL-C, TG, NT-proBNP, creatinine, LAD, LVID, and LVEF.

CI, confidence interval; OR, odds ratio. The bold values indicate statistically significant *P*-values (*P* < 0.05).

**Figure 2:**
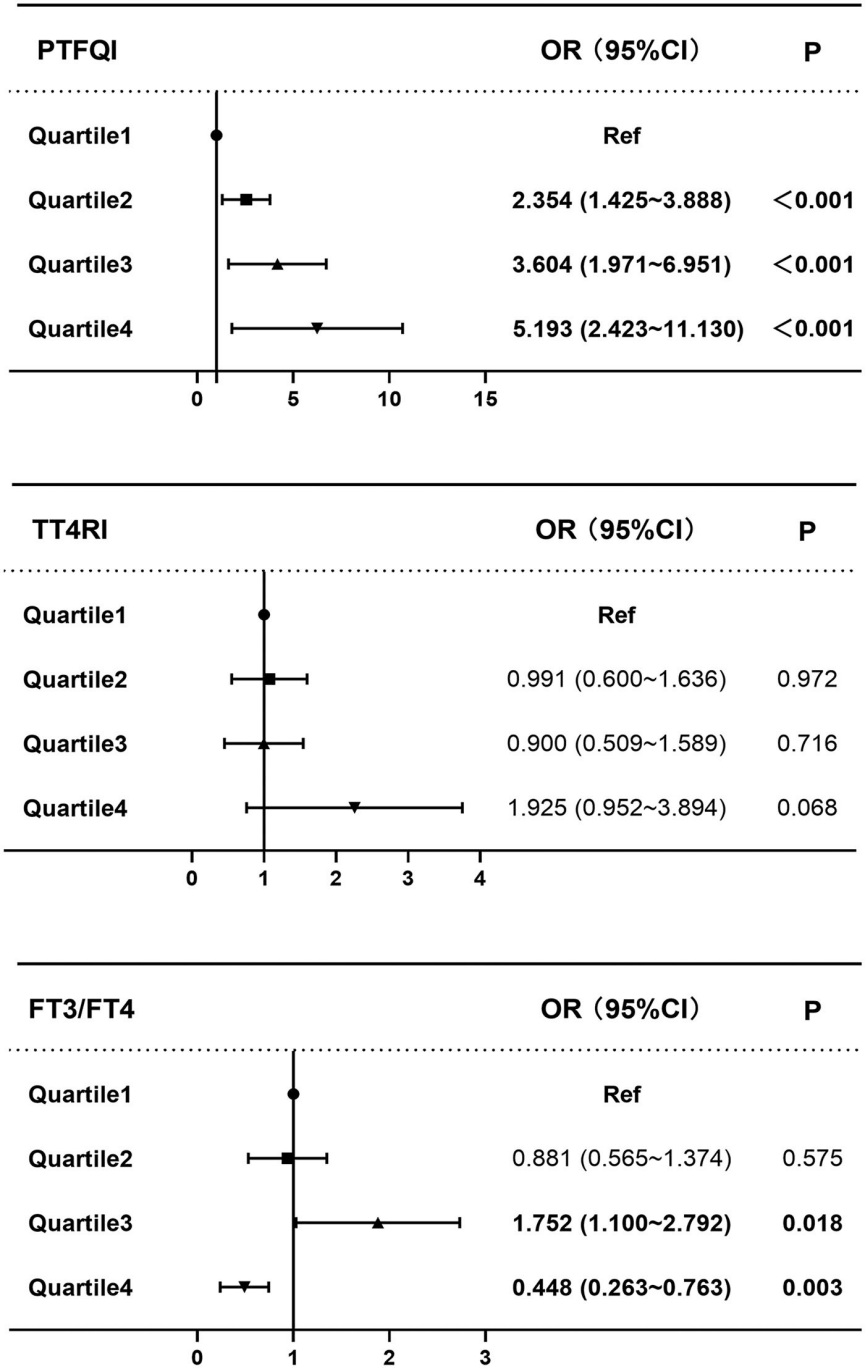
Forest plots of the ORs for TT4RI, PTFQI, and FT3/FT4 ratio, with Q1 as the reference. The ORs were adjusted for age, sex, smoking, alcohol consumption, hypertension, diabetes mellitus, stroke, coronary artery disease, heart failure, LDL-C, HDL-C, TG, NT-proBNP, creatinine, LAD, LVID, and LVEF. CI, confidence interval.

Quartile analyses of the thyroid hormone sensitivity indices yielded consistent findings (Table 3 and Figure 2). In Models 1 and 2, at the Q4 level, the FT3/FT4 ratio was significantly and inversely associated with persistent AF (Model 1: OR 0.299, 95% CI: 0.199–0.450; Model 2: OR 0.301, 95% CI: 0.196–0.464; both *P* < 0.001). In Model 3, compared with the lowest quartile (Q1), the second quartile (Q2) was not significantly associated with persistent AF (OR 0.881, 95% CI: 0.565–1.374, *P* > 0.05). Interestingly, Q3 was associated with a significantly increased risk (OR 1.752, 95% CI: 1.100–2.792, *P* for trend = 0.018), while Q4 was significantly associated with a reduced risk (OR 0.448, 95% CI: 0.263–0.763, *P* = 0.003), indicating a non-linear relationship. For the PTFQI, a dose-response relationship was observed, with a progressively increasing risk of persistent AF across higher quartiles. In Model 3, the ORs (95% CIs) for Q2, Q3, and Q4 were 2.354 (1.425–3.888), 3.604 (1.971–6.951), and 5.193 (2.423–11.130), respectively (all *P* < 0.001). Conversely, TT4RI was significantly associated with persistent AF in Q4 in Models 1 and 2 (Model 1: OR 3.051, 95% CI: 1.751–5.315; Model 2: OR 3.103, 95% CI: 1.759–5.472; both *P* for trend <0.001). However, this association was no longer statistically significant after full adjustment in Model 3.

**Table 3:** Logistic regression analysis for the association of thyroid hormone sensitivity and persistent AF across the quartiles of TT4RI, PTFQI, and FT3/FT4 ratio.

		Quartile2		Quartile3		Quartile4	
Variable	Quartile1	OR (95%CI)	*P*	OR (95%CI)	*P*	OR (95%CI)	*P*
FT3/FT4 ratio							
Model 1	1.000	0.737 (0.518–1.049)	0.091	0.991 (0.692–1.420)	0.961	0.299 (0.199–0.450)	<**0**.**001**
Model 2	1.000	0.754 (0.527–1.079)	0.123	1.023 (0.706–1.485)	0.903	0.301 (0.196–0.464)	<**0**.**001**
Model 3	1.000	0.881 (0.565–1.374)	0.575	1.752 (1.100–2.792)	**0**.**018**	0.448 (0.263–0.763)	**0**.**003**
TT4RI							
Model 1	1.000	0.993 (0.666–1.480)	0.971	1.075 (0.685–1.688)	0.752	3.051 (1.751–5.315)	<**0**.**001**
Model 2	1.000	0.987 (0.659–1.477)	0.948	1.101 (0.696–1.741)	0.682	3.103 (1.759–5.472)	<**0**.**001**
Model 3	1.000	0.991 (0.600–1.636)	0.972	0.900 (0.509–1.589)	0.716	1.925 (0.952–3.894)	0.068
PTFQI							
Model 1	1.000	2.179 (1.470–3.231)	<**0**.**001**	2.542 (1.591–4.062)	<**0**.**001**	3.251 (1.806–5.853)	<**0**.**001**
Model 2	1.000	2.208 (1.481–3.291)	<**0**.**001**	2.621 (1.619–4.243)	<**0**.**001**	3.178 (1.731–5.837)	<**0**.**001**
Model 3	1.000	2.354 (1.425–3.888)	<**0**.**001**	3.604 (1.971–6.951)	<**0**.**001**	5.193 (2.423–11.130)	<**0**.**001**

Model 1: Crude model. Model 2: Adjusted for sex and age. Model 3: Adjusted for age, sex, smoking, alcohol consumption, hypertension, diabetes mellitus, stroke, coronary artery disease, heart failure, LDL-C, HDL-C, TG, NT-proBNP, creatinine, LAD, LVID, and LVEF. The bold values indicate statistically significant *P*-values (*P* < 0.05).

## 4 Discussion

In this study, we comprehensively evaluated the association between thyroid hormone sensitivity and AF in patients with normal thyroid function. Elevated central thyroid hormone sensitivity indices (TT4RI and PTFQI) and reduced peripheral FT3/FT4 ratio were significantly associated with the presence of persistent AF. These associations remained robust after adjusting for potential confounding factors, including age, sex, smoking, alcohol consumption, hypertension, diabetes mellitus, stroke, CAD, heart failure, echocardiographic parameters, and multiple serum biochemical markers. These findings suggest that impaired responsiveness to thyroid hormones at the tissue level may be an important determinant in AF progressing from paroxysmal to persistent AF.

Previous studies have demonstrated a robust association between thyroid hormone levels and cardiovascular function (19–21). Both myocardial and vascular tissues express thyroid hormone receptors, and minor fluctuations in thyroid hormone levels can significantly influence cardiovascular homeostasis (22–24). Higher FT4 levels have been significantly associated with an increased risk of AF even within the euthyroid range (25–27). Our study revealed that FT4 levels, even within the normal reference range, were significantly higher in patients with persistent AF than those with paroxysmal AF. However, the association between TSH levels within the normal range and AF remains controversial. Some studies did not observe any significant relationship between normal-range TSH levels and AF risk (25, 27), whereas others suggested that lower TSH levels, even within the euthyroid range, may predispose individuals to AF (28, 29). Conversely, numerous studies have indicated that higher TSH levels within the normal range are associated with an increased risk of AF recurrence following catheter ablation (30). Our study revealed that patients with persistent AF exhibited significantly higher TSH levels, although within the normal range, than those with paroxysmal AF. This finding may indicate a compensatory increase in TSH levels, resulting from reduced peripheral sensitivity to thyroid hormones at the tissue level.

In addition to individual thyroid function markers such as FT4 and TSH, our findings revealed that elevated PTFQI and TT4RI were significantly associated with persistent AF in patients with normal thyroid function. Previous studies have suggested that reduced thyroid hormone sensitivity is more prevalent than overt thyroid hormone resistance in the general population and is closely linked to metabolic disorders (31, 32). Sensitivity indices, such as PTFQI, TT4RI, TFQI, and TSHI, have been associated with the development of obesity, metabolic syndrome, hypertension, diabetes mellitus, and their related complications (31, 33–36). Alonso-Ventura et al. conducted a cross-sectional study in a Spanish population about arrhythmias. This study reported that PTFQI was not only significantly associated with diabetes mellitus, ischemic cardiomyopathy, and hypertension but also positively correlated with the prevalence of AF, which increased with increasing PTFQI levels (16). Similarly, PTFQI and FT4 levels were significantly elevated in patients with AF compared to those in the general population with normal thyroid function (15). Our study further revealed that the PTFQI and TT4RI levels were significantly higher in patients with persistent AF than in those with paroxysmal AF, even within the AF population. These findings suggest that impaired thyroid hormone sensitivity may contribute to the onset of AF and may be involved in its progression, highlighting its potential value for clinical risk assessment. However, inconsistent results have been reported in other populations. For instance, the RCSCD-TCM study, which included over 28,000 patients with CAD, reported that patients with arrhythmia exhibited significantly lower PTFQI, TFQI, TT4RI, and TSHI levels than healthy controls. Logistic regression also demonstrated negative correlations between these indices and arrhythmia (37). This discrepancy may be attributed to variations in the study population. The RCSCD-TCM study included a broad spectrum of arrhythmias, including AF, atrial flutter, and conduction block, whereas our study focused exclusively on AF and further distinguished between the paroxysmal and persistent subtypes. This refined classification may identify the potential role of thyroid hormone sensitivity in AF progression. Furthermore, although TT4RI was significantly associated with persistent AF in the univariate and partially adjusted models, its quartile-based associations lost statistical significance after full adjustment (Model 3), indicating reduced robustness in complex clinical contexts. Conversely, the PTFQI maintained consistent significance across all models, thereby supporting its potential use in clinical evaluation and risk stratification.

The FT3/FT4 ratio reflects peripheral sensitivity to thyroid hormones and represents the efficiency of T4 conversion to biologically active T3 in peripheral tissues. A lower FT3/FT4 ratio indicates reduced peripheral thyroid hormone sensitivity (38). Previous studies have reported that a decreased FT3/FT4 ratio is significantly associated with diabetic peripheral neuropathy (35), and serves as an independent predictor of all-cause and cardiovascular mortality in patients with heart failure (39). Conversely, higher FT3/FT4 ratios may produce cardioprotective effects in patients with acute myocardial infarction by improving myocardial energy metabolism and reducing oxidative stress and fibrosis (40). In a study that included patients with heart failure with preserved ejection fraction, those with AF often exhibited a reduced FT3/FT4 ratio (41). Consistent with these findings, our study demonstrated that a lower FT3/FT4 ratio is significantly associated with persistent AF. Notably, quartile analysis revealed a non-linear relationship between the FT3/FT4 ratio and the risk of persistent AF. The risk peaked in Q3 but decreased in Q4. This pattern suggests a potential threshold or moderating mechanism that influences the predictive value of FT3/FT4. Interestingly, a Mendelian randomization analysis reported that individuals with elevated FT3/FT4 ratios exhibited a significantly increased risk of AF (42). This discrepancy may be attributed to differences in the study design. Our study focused on differences in thyroid hormone sensitivity indices between paroxysmal and persistent AF. Future large-scale prospective studies are required to validate this potential non-linear association and to explore the underlying mechanisms. Nevertheless, the present findings provide cross-sectional evidence supporting an association between impaired thyroid hormone sensitivity and AF type, offering a basis for future prospective investigations.

## 5 Limitations

Our study has several limitations. First, causality between thyroid hormone sensitivity indices and AF type could not be established because of the cross-sectional nature of the study, and longitudinal follow-up data were not systematically collected, precluding evaluation of their prognostic value. Second, the study population consisted of Chinese patients from a single center, which may limit the generalizability of the findings to populations with different ethnic or geographic backgrounds. Third, our study did not include thyroid hormone sensitivity indices such as TSHI and TFQI, which have been used in previous studies to evaluate central feedback regulation. This decision was made to maintain the stability of the model, as TSH data in our cohort exhibited significant bias, and these indices exhibited strong collinearity with PTFQI and TT4RI. While this limits comparison with existing literature, we believe that the inclusion of PTFQI, TT4RI, and the FT3/FT4 ratio sufficiently determines the association between thyroid hormone sensitivity and AF type. Finally, this study did not exclude patients taking thyroid-modulating medications such as amiodarone. Although all participants underwent thyroid function testing and those with clinical or subclinical thyroid dysfunction were excluded, we cannot entirely rule out the possibility that such medications may have subtle effects on tissue-level thyroid hormone sensitivity within the normal range. This potential residual effect may have had a minor influence on our findings.

## 6 Conclusions

In conclusion, our study demonstrated that elevated PTFQI and TT4RI and a decreased FT3/FT4 ratio were significantly associated with persistent AF in euthyroid patients with AF. These associations remained independent after adjustment for multiple clinical and echocardiographic factors. These findings suggest that impaired central and peripheral thyroid hormone sensitivity may be associated with a more advanced AF phenotype and provide preliminary evidence for the potential utility of these indices in AF risk stratification. However, their precise clinical utility requires further validation in large-scale prospective studies.

## Data Availability

The data supporting the findings of this study are available from the corresponding authors upon reasonable request.
